# Ag_3_Sn Compounds Coarsening Behaviors in Micro-Joints

**DOI:** 10.3390/ma11122509

**Published:** 2018-12-10

**Authors:** Ye Tian, Ning Ren, Zhihua Zhao, Fengshun Wu, Suresh K. Sitaraman

**Affiliations:** 1 School of Mechanical and Electrical Engineering, Henan University of Technology, Zhengzhou 450052, China; ningren001@126.com (N.R.); zzuzhaozhihua@gmail.com (Z.Z.); 2 George W. Woodruff School of Mechanical Engineering, Georgia Institute of Technology, Atlanta, GA 30332, USA; 3 School of Materials Science and Engineering, Huazhong University of Science and Technology, Wuhan 430074, China; fengshunwu@hust.edu.cn

**Keywords:** micro-joints, Ag_3_Sn compounds, coarsening kinetics, reliability, flip chip assemblies

## Abstract

As solder joints are being scaled down, intermetallic compounds (IMCs) are playing an increasingly critical role in the reliability of solder joints, and thereby an in-depth understanding of IMCs microstructure evolutions in micro-joints is of great significance. This study focused on coarsening behaviors of Ag_3_Sn compounds in Sn-3.0Ag-0.5Cu (SAC305) micro-joints of flip chip assemblies using thermal shock (TS) tests. The results showed that the Ag_3_Sn compounds grew and rapidly coarsened into larger ones as TS cycles increased. Compared with such coarsening behaviors during thermal aging, TS exhibited a significantly accelerating influence. This predominant contribution is quantitatively determined to be induced by strain-enhanced aging. Moreover, based on observations for Ag_3_Sn microstructure evolutions during TS cycling, one particular finding showed that there are two types of coarsening modes (i.e., Ostwald ripening and Necking coalescence) co-existing in the Ag_3_Sn coarsening process. The corresponding evolutions mechanism was elucidated in a combination of simulative analysis and experimental validation. Furthermore, a kinetic model of the Ag_3_Sn coarsening was established incorporating static aging and strain-enhanced aging constant, the growth exponent (*n*) was calculated to be 1.70, and the predominant coarsening mode was confirmed to be the necking coalescence.

## 1. Introduction

In electronic devices, solder joints generally provide electrical and thermal connections among different components and various circuits, and are regarded as the weakest link in advanced packages of electronic devices [1,2]. Recently, environmental issues and governmental legislation have led to an increased need to implement lead-free solder. In order to achieve this, SnAgCu solders have been recommended as the most promising candidates to replace conventional SnPb solders in microelectronics [3,4]. This is due to their good mechanical properties, adequate wettability, and acceptable melting temperature. Among these SnAgCu solders, the Sn-3.0Ag-0.5Cu (SAC305) alloy has become the benchmark solder as it can be utilized in a wide array of electronic devices [5,6].

In SnAgCu solders, Ag atoms can enhance the thermo-mechanical behaviors and strain rates of the mechanical performance. This is because a large number of finely dispersed Ag_3_Sn particles, that form by the reaction of Ag and liquid Sn in the reflowing process, restrain the deformation and grain recrystallization of β-Sn subjected to external stressing [7,8]. However, during both storage and service, the microstructure of the SnAgCu solder joints, consisting of β-Sn rich zones, Ag_3_Sn, and Cu_6_Sn_5_ compounds, is susceptible to coarsen over time [9]. The resulting large Ag_3_Sn particles tend to weaken the mechanical properties of solder joints, thereby degrading their thermal fatigue lifetime [10,11]. It is worth noting that coarsening impacts of Ag_3_Sn compounds on the mechanical properties are much greater, relative to Cu_6_Sn_5_ compounds, owing to their higher volume proportion in the solder joint [12,13]. More significantly, as the size of solder joints scales down, so as to meet miniaturization and multi-functions of electronic devices, the volume proportion of intermetallic compounds (IMCs) in the solder joint has dramatically increased, playing a critical role in joint reliability [14,15]. Hence, the coarsened Ag_3_Sn particles are expected to exhibit a greater negative impact on the reliability of micro-joints. Consequently, a precise understanding of Ag_3_Sn coarsening behaviors is of great significance to further evaluate and enhance the reliability of micro-joints.

To date, some research has been conducted into the coarsening behaviors of Ag_3_Sn compounds over time [16,17]. Most of studies placed their emphasis on thermal aging influences, and found that Ag_3_Sn coarsening is visible as the aging time increased, indicating that such coarsening behaviors follow the Ostwald ripening principle [13,18]. It is worth noting that the reliability of solder joints with large Ag_3_Sn particles are significantly sensitive to thermal cycling loading, since thermally induced stresses commonly concentrate at the interface of the solder matrix, and large Ag_3_Sn particles [19]. This can induce a crack occurrence at the interface, and markedly debase the reliability of solder joints, especially for micro-joints that undergo larger stress [20]. Thermo-mechanical failure of solder joints has recently been reported to be a severe reliability issue in advanced packages [21,22]. However, thus far, considerably few investigations have concentrated on Ag_3_Sn coarsening behaviors in SnAgCu solder joints subjected to thermal cycling loading, especially for SnAgCu micro-joints, and the relevant coarsening mechanisms are not well understood. Qi et al. used simulated samples with two large solder joints to investigate thermal-shearing influences on Ag_3_Sn coarsening. They observed that thermal shearing can accelerate Ag_3_Sn coarsening by comparing this to thermal aging. Furthermore, they presumed that this coarsening phenomenon may be induced by both static aging and strain-enhanced aging in SnAgCu solder joints [23]. Liang et al. found that Ag_3_Sn compounds exhibited more evident coarsening at higher strain regions of the solder joint during thermal cycling, indicating that strain is capable of promoting Ag_3_Sn coarsening [24]. Dutta et al. systematically investigated Ag_3_Sn coarsening behaviors during thermo-mechanical cycling, and revealed that the cumulative strains induced by the ramp time of one cycle, can coarsen Ag_3_Sn particles. Moreover, they also presented coarsening kinetics based on Ag atomic bulk diffusion [25,26].

Apart from the above descriptions, there is little in the literature that has reported Ag_3_Sn coarsening in solder joints under thermo-mechanical conditions, and referred to this as having an impact on coarsening behaviors that likely depend on strain-enhanced aging. The contribution of strain-enhanced aging to the Ag_3_Sn coarsening in the micro-joint has not been evaluated quantitatively under TS cycling. Simultaneously, the microstructure evolutions of Ag_3_Sn compounds in the coarsening process are not understood well. Accordingly, this lack of understanding does not provide much insight into the physical mechanisms of microstructure coarsening that occurs during thermal cycling. Furthermore, it is essential to establish an adaptive microstructure coarsening model to predict such coarsening behaviors in SnAgCu solder joints. In particular, for the micro-joints in advanced packages, Ag_3_Sn coarsening is anticipated to become more complicated, due to faster variations of the solder composition, higher thermally induced stresses, and stronger cross-interactions between different metal pads [27,28].

The objective of our research was to systematically investigate coarsening behaviors of Ag_3_Sn compounds in SAC305 micro-joints of flip chip assemblies using thermal shock (TS) tests. Strain-enhanced influence on the Ag_3_Sn coarsening was quantified to analyze their size by comparing variations at the different intervals of TS cycling and thermal aging. Combined microstructure observations with finite element simulative analysis were conducted and Ag_3_Sn microstructure evolutions in the coarsening process are elucidated in detail. Moreover, a kinetic model was established to predict coarsening behaviors that incorporates both static aging and strain-enhanced aging constant. Moreover, we discuss in detail the predominant coarsening mode during TS cycling. 

## 2. Experimental Procedure

The samples used in this study were 10 mm × 10 mm silicon flip chips with a peripheral row of 376 solder bumps at 100 µm pitch and 60 µm diameter, which is a common type used in fine-pitch flip chip assembly. The composition of the solder alloy was Sn-3.0Ag-0.5Cu (wt.%) (SAC305). The flip chips with Ni pads were assembled onto organic substrates with Cu pads by using a standard lead-free thermal reflow profile, i.e., a peak temperature of 240 °C with dwelling approximately 60 s above the solder melting point (217 °C). After cooling, the assemblies were underfilled with an epoxy-based underfill, and cured at 160 °C for 7 min. The electrical resistance of daisy chains was measured to determine assembly quality. The samples were subjected to air-to-air TS testing from −55 °C to 125 °C according to MIL-STD-883H Method 1010.8 Test Condition B, which is a common and preferred way to evaluate the reliability of microelectronic products. Four specimens were extracted at each stage of 0, 400, 800, 1200, and 2000 cycles. Another set of specimens were isothermally aged at 125 °C for up to 500 h in a standard convection oven. Four specimens were extracted at each stage of 0, 100, 200, 300, and 500 h. All specimens were cold-mounted in acrylic, polished down to a 0.05 µm finish, and then etched for 5 s using 5%HCl-95%C_2_H_5_OH solution. Backscattered electron (BSE) mode in a scanning electron microscope (SEM, Ultra-60, Zeiss, Oberkochen, Germany) was used to observe the microstructure morphology of the solder joints, and energy-dispersive x-ray spectrometry (EDS, 50mm-SDD, Oxford, Abingdon, UK) was used to characterize the composition and elemental distribution of the IMCs. Image analysis software (MEDIA CYBERNETICS, Oxon, UK) was used to measure and calculate the IMCs size in the solder matrix. Microstructure observations were focused on corner solder joints, since they are recognized as the most critical solder joints of flip chip assemblies due to experience the most strains and stresses during TS cycling [29].

## 3. Finite Element Analysis Procedure

**Figure 1:**
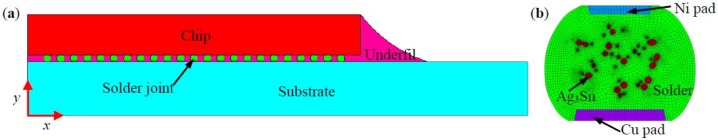
Geometry model and finite element (FE) model of flip ship assembly: (**a**) Geometry model of the assembly; (**b**) FE model of the corner joint.

To further comprehend the microstructure evolutions of the Ag_3_Sn compounds in the coarsening process, finite element (FE) simulation was conducted to analyze the stress distribution in the corner micro-joint under TS conditions. Considering extremely complicated structures of the micro-joint with irregular morphological IMCs, a two-dimensional model was built to avoid considerable computational expense in this study. A geometry model of the flip chip assembly is presented in Figure 1a. According to the geometric symmetry of this assembly, one-half of this modeling was set up. As shown in Figure 1b, the corner joint was modeled fully since it was anticipated that it would undergo the highest stresses. The others were modeled without considering the IMCs structures to reduce the elements number, and hence the computational time. Based on the IMCs morphological characteristics, the round-shaped structure was used to emulate the Ag_3_Sn particles into the solder matrix. The FE model is composed of 154,946 quadrilateral elements and 473,282 nodes. Symmetric boundary conditions were defined on the symmetry axis (y axis) of the assembly, and the bottom node at the symmetry axis was completely fixed to prevent free body motion. The stress-free temperature of the solder was assumed to be the melting temperature (220 °C). All the other materials were assumed to be stress-free at 160 °C, which is the underfill curing temperature. The entire assembly was simulated to be cooled down to the room temperature and then subjected to the TS temperature profile. Additional details can be found in [29].

During TS cycling, Sn based solders demonstrated significant viscoplastic deformation owing to high homologous temperature [15]. Anand’s model was used as a unified viscoplastic model can accurately describe viscoplastic behaviors of SAC solders [30]. Therefore, Anand’s model was adopted in this study to simulate viscoplastic deformation behaviors of SAC305 solder, while all other materials were assumed to be linearly elastic. Based on FE theory, element integration point stresses are commonly described by generalized Hooke’ law, {*σ*} = [*D*] {*εel*}, where {*σ*} is the stress vector, [*D*] is the elasticity matrix and its value depends on elastic modulus and Poisson’s ratio of materials, and {*εel*} refers to elastic strains. For linearly elastic materials, {*εel*} = {*ε*} − {*εth*}, where {*ε*} is total strain vector, and *{εth}* thermal strain vector. The thermal strain vector can be described as: {*εth*} = Δ*T* [*αx αy αz* 0 0 0]*^T^*, where Δ*T* represents the temperature difference during TS cycling, *αx*, *αy*, and *αz* are coefficient of thermal expansion (CTE) in *x*, *y*, and z direction, respectively. For the case of viscoplastic materials, the definition of elastic strain is given by the form of: {*ε_el_*} = {*ε*} − {*ε_th_*} − {*ε_vp_*}, where *ε_vp_* is viscoplastic strain vector. According to Anand’s model, the viscoplastic strain rate is described by a flow equation and three evolution equations [15]:

Flow equation:

(1) $${\dot{\varepsilon }}_{vp}=A{\left[\sinh\left(\frac{\xi \sigma }{s}\right)\right]}^{1/m}\exp\left(\frac{-Q}{kt}\right)$$

Evolution equations:

(2) $$\dot{s}=\left\{h_{0}{\left(\left|B\right|\right)}^{a}\frac{B}{\left|B\right|}\right\}{\dot{\varepsilon }}_{vp}$$

(3) $$B=1-\frac{s}{s^{*}}$$

(4) $$s^{*}=\hat{s}{\left[\frac{{\dot{\varepsilon }}_{vp}}{A}\exp\left(\frac{-Q}{kt}\right)\right]}^{n}$$

where the nine material constants of *A*, *Q/k*, *ξ*, *m*, $\hat{s}$, *n*, *h*_0_, *a*, *s*_0_ are described in the literature [31], and these constants for SAC305 solder in our FE model can be obtained from the same literature. Furthermore, it was assumed that the CTE of the different materials in our model were all homogeneous and isotropic. The linearly elastic material properties for Ni, Cu, and Ag_3_Sn are provided in Table 1, and the others can be found in our previous studies [32].

## 4. Results and Discussion

### 4.1. Coarsening Characteristic of Ag_3_Sn Compounds during TS Cycling

To definitively elucidate TS cycling effects on Ag_3_Sn coarsening behaviors, the concept of effective thermal aging time (denoted as *t_eff_*) was employed to compare the results against isothermal aging. Under the TS conditions in this study, the specimens were dwelled for 15 min at both 125 °C and −55 °C with a 5 s transition time, the diffusion rate of Ag atoms is so low at −55 °C in Sn based solders that it is practically negligible to affect Ag_3_Sn growth [23]. Consequently, based on the formula of *t_eff_* (units of s) = *t_top_* × *N*, where *t_top_* (units of s) is the dwell time at high temperature extreme, and *N* is the number of elapsed cycle, the specimens would be kept at 125 °C for 100 h under 400 TS cycles, and the magnitude of such Ag_3_Sn size could be compared against that determined at 100 h using the isothermal aging at 125 °C. Similarly, 300 and 500 h could be used as the effective time for 1200 and 2000 TS cycles. Such an effective thermal aging time has been previously used to investigate the interfacial IMCs growth subjected to thermal cycling loading [23,32].

**Figure 2:**
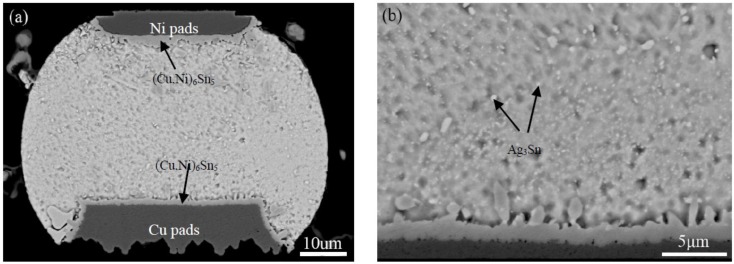
Across-sectional back-scattered electron (BSE) image of the corner SAC305 micro-joint in flip chip assembly after assembly reflow: (**a**) Entire solder joint; (**b**) local magnified image around Ni pad.

**Figure 3:**
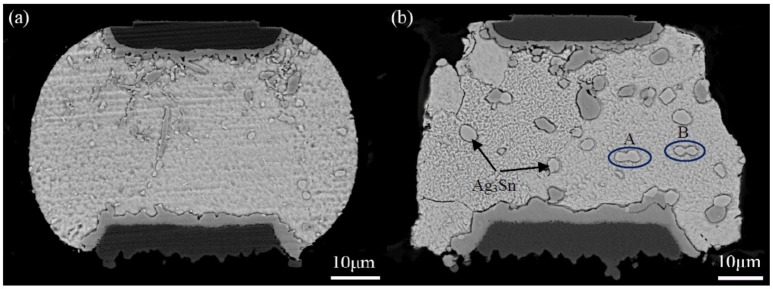
Cross-sectional BSE images of SAC305 micro-joints in flip chip assembly: (**a**) After 500 h aging time; (**b**) after 2000 thermal shock (TS) cycles.

**Figure 4:**
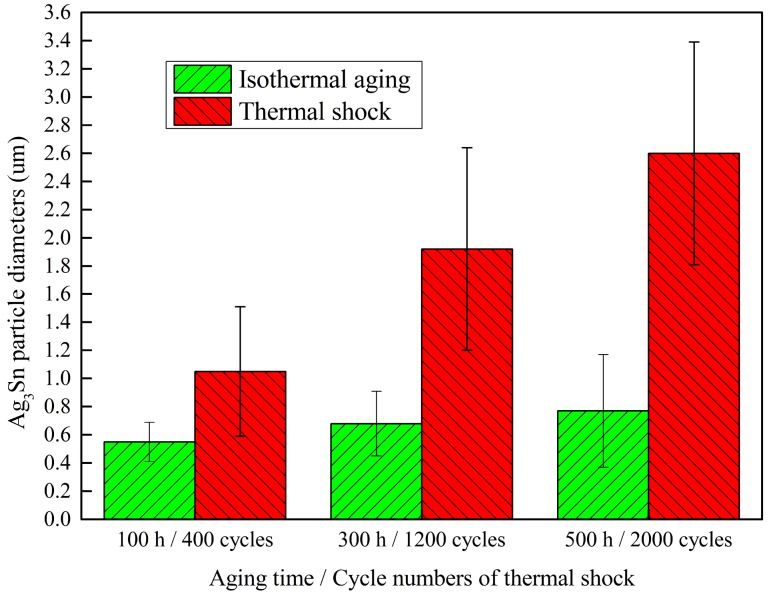
Contribution of the strain-enhanced aging to the Ag_3_Sn coarsening during TS cycling.

Figure 2 shows the cross-sectional back-scattered electron (BSE) image of the corner micro-joint after assembly reflow. As seen in Figure 2a, the micro-joint is well shaped with an oval-like shape, and firmly bonded to both the Ni pad of the chip side and Cu pad of the substrate side. Based on the BSE contrast, two types of the IMCs were observed to form at both pad interfaces, as seen in Figure 2a,b. Results of EDS analysis were used to identify the IMCs identity, as presented in Table 2. The dark IMCs, with irregular layer-shape, appeared at the Ni and Cu pad interface (Cu,Ni)_6_Sn_5_. A great number of light particle-shaped IMCs dispersed in the solder matrix were Ag_3_Sn. The cross-sectional BSE images of the corner micro-joints after 500 h aging time and 2000 TS cycles are shown in Figure 3. As we expected, the microstructure evolutions of Ag_3_Sn compounds displayed an identical trend under these two conditions. Fine Ag_3_Sn particles became larger in size but fewer in number upon aging time and thermal cycles. However, one discrepancy was still clear, namely that the TS cycling affected more greatly on coarsening behaviors relative to the thermal aging, and thereby exhibiting an effect on the acceleration. Ag_3_Sn coarsening was reported to depend mainly on both strain-enhanced aging and static aging under thermo-mechanical conditions. Therefore, in the current study, strain-enhanced aging can be responsible for the above-mentioned coarsening acceleration. To further quantify the contribution of strain-enhanced aging to Ag_3_Sn coarsening during TS cycling, the average diameter of the Ag_3_Sn particles after isothermal aging and TS cycling, denoted as *D_iso_* and *D_TS_*, was measured and calculated using digital software. The corresponding data are plotted in Figure 4, it is evident that strain-enhanced aging has the predominant contribution to this coarsening. The contribution rate was calculated to reach as high as above 70% after 2000 cycles according to the formula of (*D_TS_* − *D_iso_*)/*D_TS_* × 100. As a consequence, strain-enhanced aging was confirmed to dominate such coarsening behaviors in the SAC305 micro-joints of flip chip assembly when subjected to TS cycling.

### 4.2. Coarsening Mechanisms of Ag_3_Sn Compounds

**Figure 5:**
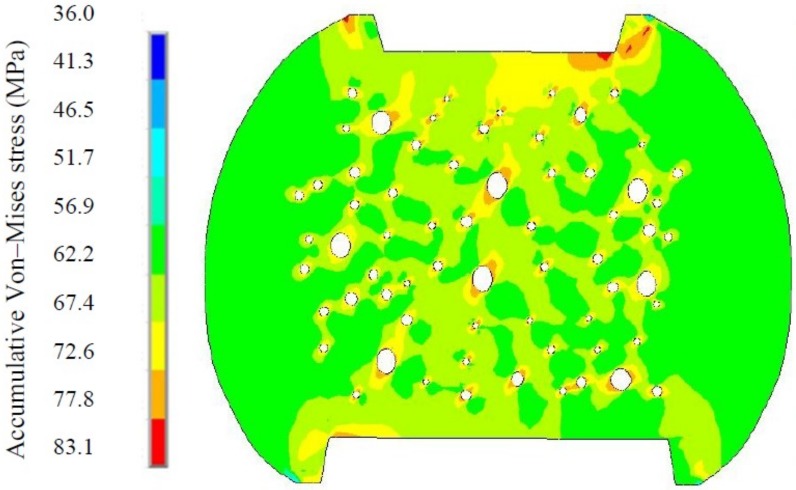
An accumulative Von-Mises stress contour of the corner micro-joint with dispersed Ag_3_Sn particles in different size after eight TS cycles.

From the viewpoint of Ag_3_Sn particles transition in size and number, Ag_3_Sn coarsening behaviors should adhere to the Ostwald ripening principle. This principle is defined as the growth of larger crystals at the expense of smaller ones with the higher interfacial enthalpy, and the driving force of this process originating from the reduction of the total interfacial energy. In the present study, based on this principle, the Ag_3_Sn coarsening process can be reasonably described as small Ag_3_Sn particles being dissolved to release Ag atoms, which diffused through the solder matrix to the region around the large ones, and then re-precipitated to facilitate the further growth of the large ones during thermal aging and TS cycling. Furthermore, to explain the extremely remarkable coarsening of the Ag_3_Sn particles during TS cycling, finite element simulation was employed to analyze the stress distribution in the micro-joints, aiming to correlate it with this phenomenon. Figure 5 shows an accumulative Von-Mises stress contour of the corner micro-joint after eight TS cycles. It is clear that the greater stress mainly distributed around the Ag_3_Sn particles, these stressed regions appear to form a “stress network” that surrounds the Ag_3_Sn particles. As is well known, stress is capable of producing substantial crystal defects, such as dislocations and vacancies. These defects can serve as rapid diffusion pathways to drive the atoms diffusion in the solder matrix. Furthermore, existing literature has reported that the volume diffusion of reaction atoms is a governing mechanism for the second phase coarsening that follows the Ostwald ripening principle [25]. Based on these two points, the “stress-network” in the solder matrix seems to provide a fast pathway to accelerate the dissolved Ag atoms diffusion from the smaller Ag_3_Sn towards the larger ones, thereby leading to the coarsening under thermal cycling conditions.

**Figure 6:**
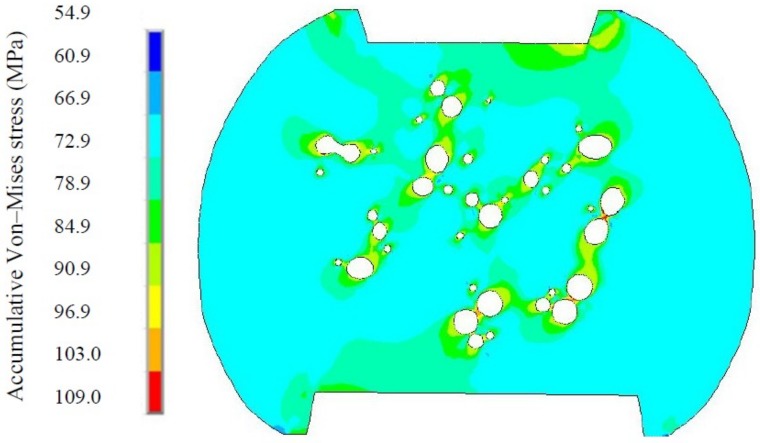
Accumulative Von-Mises stress contour of the micro-joint with adjacent and similar-size large Ag_3_Sn particles after eight TS cycles.

**Figure 7:**
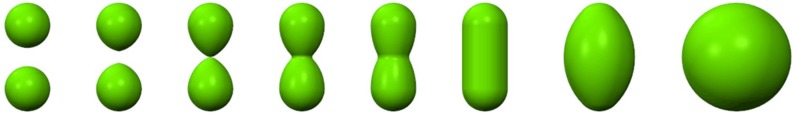
A schematic diagram of the Ag_3_Sn coarsening following the necking-coalescence during TS cycling.

**Figure 8:**
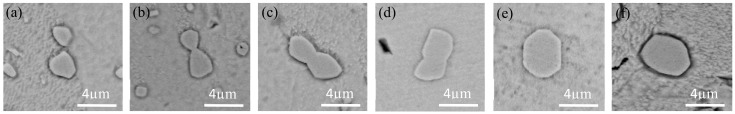
Microstructure evolutions of the large Ag_3_Sn particles following necking-coalescence: (**a**) the adjacent particles protruding towards each other; (**b**) two large particles bridged by a necking; (**c**–**f**) the necking-shaped Ag_3_Sn evolves to the uniform curvature.

Interestingly, an extraordinary morphology of the coarsened Ag_3_Sn particles attracted our attention, marked as A and B in Figure 3. Two adjacent large Ag_3_Sn particles, with nearly similar size, were observed to come into contact in the shape of a necking, indicating that they are likely to directly coalesce during TS cycling, named as necking-coalescence. Due to only the presence of this coarsening characteristic during TS cycling, the thermally induced stress was naturally taken into account to be the main cause. Figure 6 shows an accumulative Von-Mises stress contour of the corner micro-joint with adjacent and similar-size large Ag_3_Sn particles after eight TS cycles. The stress concentration appeared on the spaces between the neighboring large Ag_3_Sn particles. Accordingly, it is reasonable to suppose that the atoms dissolved from the small Ag_3_Sn particles prefer to diffuse to these higher stressed regions, and then locally pre-precipitated at the larger Ag_3_Sn particles until it bridged them. This can force a necking to take place in the coarsening process. As the necking formed, remarkable curvature gradients with strong localization at the necking can drive the migration of Ag_3_Sn grain boundaries, resulting in the establishment of a single particle with uniform curvature at the interface, ultimately a spherical large particle. The detailed process of the necking-coalescing coarsening was drawn in Figure 7. Our experimental results were able to validate such a coarsening process, as seen in Figure 8. It mainly comprises of: The local position of the adjacent particles protruding towards each other; two large particles bridged by a necking; and the necking-shaped Ag_3_Sn evolves to the uniform curvature at the interface.

### 4.3. Coarsening Kinetics of Ag_3_Sn Compounds during TS Cycling

In this section, the emphasis is placed on coarsening kinetics of Ag_3_Sn compounds under TS conditions, aiming to further elucidate the Ag_3_Sn coarsening mechanism by developing a coarsening kinetic model, and to determine the predominant coarsening mode. The effective aging time (*t_eff_*) mentioned in Section 4.1 was introduced into the growth kinetic model of the second phase under isothermal aging [34], and the Ag_3_Sn coarsening kinetic model during TS cycling can be expressed as follows:

(5) $$D^{n}-D_{0}^{n}=k_{0}t_{eff}\exp\left(\frac{-Q}{RT_{top}}\right)=k_{0}t_{top}N\exp\left(\frac{-Q}{RT_{top}}\right)$$

where *D* and *D*_0_ (units of m) represent the average diameter of the Ag_3_Sn particles at N and 0 cycles individually, *n* is the growth exponent, and its value depends on the rate-controlling mechanism, and *n* = 2 represents the solute atoms transfer across the particle/matrix interface, while *n* = 3 corresponds to the volume diffusion [35,36]; *k*_0_ is the static aging coarsening kinetics constant, *Q* (units of J/mol) is the activation energy of the relevant diffusion process, and *Q =* 69 × 10^3^ J/mol for diffusion control mechanism [37], *R* is the universal gas constant, *T_top_* (units of K) is the absolute temperature at a high temperature extreme.

As aforementioned, the Ag_3_Sn coarsening depends on the combined action of the static aging and strain-enhanced aging during TS cycling. Therefore, these two factors were incorporated into this constitutive law. In terms of the model proposed by Dutta’ investigation, $2M\varphi \dot{\gamma }t_{ramp}N$, that denoted as ∆*t*, was introduced to reflect the impact of the strain-enhanced aging on the Ag_3_Sn coarsening [38]. Combining ∆*t* with Pang’ concept of the equivalent aging time (denoted as *t_eq_, t_eq_* = *t_eff_* + ∆*t*) [39], *t_eff_* can be replaced by *t_eq_* in Equation (5). Accordingly, Equation (5) was revised as Equation (6) as follows:

(6) $$D_{}^{n}-D_{0}^{n}=k_{0}\left(t_{top}+2M\varphi \dot{\gamma }t_{ramp}\right)N\exp\left(\frac{-Q}{RT_{top}}\right)$$

where *M* (units of s) is a kinetic constant representing the strain-enhanced coarsening, *ϕ* is the ratio of plastic strain to total strain imposed during the ramp, and *ϕ* ≈ 1 [26], $\dot{\gamma }$ (units of s^−1^) = *γ*/*t_ramp_* is the shear strain rate imposed on the joint during each temperature ramp of TS, *γ* is the shear strain and can be given in [40], *t_ramp_* (units of s) is the up or down ramp time during one cycle.

To determine magnitude of coarsening kinetic parameters, including the growth exponent (*n*) and strain-enhanced coarsening kinetics constant (*M*), Equation (6) was converted into Equation (7) as follows:

(7) $$\ln\frac{dD}{dN}=\ln\left(\frac{k_{0}}{n}\left(t_{top}+2M\varphi \dot{\gamma }t_{ramp}\right)\exp\left(\frac{-Q}{RT_{top}}\right)\right)+(1-n)\ln D$$

**Figure 9:**
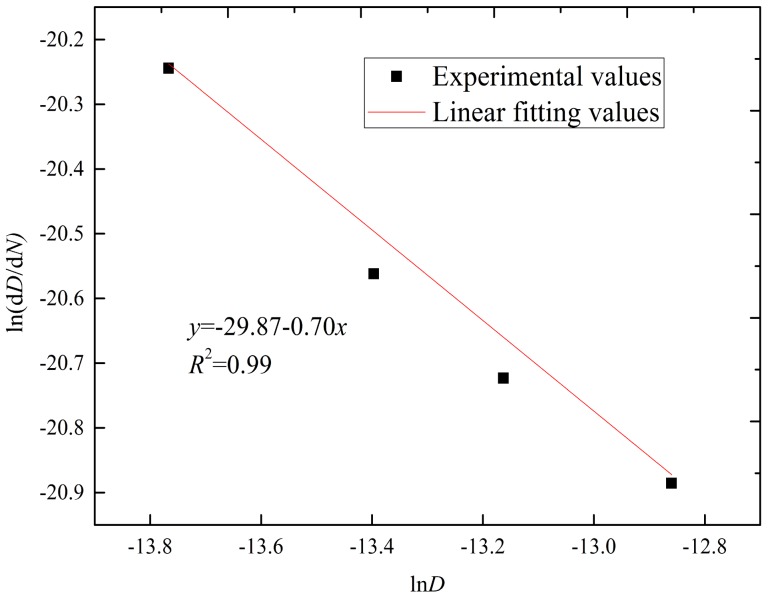
ln-ln plot of d*D*/d*N* versus *D*.

In terms of Equation (7) and the Ag_3_Sn mean particle diameter at 400, 800, 1200, and 2000 TS cycles, ln (*dD*/*dN*) versus ln*D* is plotted in Figure 9 to calculate the growth exponent (*n*) and strain-enhanced coarsening kinetics constant (*M*). The values of (1 − *n*) and $\ln\left(\frac{k_{0}}{n}\left(t_{top}+2M\varphi \dot{\gamma }t_{ramp}\right)\exp\left(\frac{-Q}{RT_{top}}\right)\right)$ can be obtained from the slope and intercept of the linear regression, respectively. Based on such a fitting, the value of *n* was determined as 1.70. Once *n* was obtained, the value of *k*_0_ was calculated as 2.88 × 10^−10^ m^2^/s according to Equation (5) combined with the mean diameter of the Ag_3_Sn particles. These data were measured and further calculated using the samples at 125 °C for 0, 100, 200, 300, and 500 h. Afterwards, the *M* value was determined as 3.49 × 10^6^ s, since the values of *k*_0_, *n*, *t_top_*, *M*, $\dot{\gamma }$, *t_ramp_* and *T_top_* are constant for a constant TS condition and joints structure. Obviously, the *n* value is close to 2, suggesting that the Ag_3_Sn coarsening behaviors were mainly attributed to the solute atoms transfer across the particle/solder matrix interface. This can confirm the necking-coalescence to be the dominant coarsening mode for the Ag_3_Sn coarsening under TS conditions.

## 5. Conclusions

This study systematically investigated Ag_3_Sn coarsening behaviors of the SAC305 micro-joint in flip chip assemblies using TS tests. The results revealed the following conclusions:

1. The Ag_3_Sn particles grew and rapidly coarsened into the larger ones as TS cycles prolonged. Such coarsening behaviors are extremely evident during TS cycling compared to thermal aging. The influential factors consisted of the static aging and strain-enhanced aging in this coarsening process, and the latter was determined to have the predominant contribution through quantitative analysis.
2. There are two types of coarsening modes co-existing in the Ag_3_Sn coarsening process during TS cycling, i.e., the Ostwald ripening and the Necking-coalescence. The presence of the necking-coalescence is mainly attributed to the higher thermally induced stress at the spaces between neighboring large Ag_3_Sn particles, and curvature gradients of bridged Ag_3_Sn particles.
3. A kinetic model was established to predict the Ag_3_Sn coarsening in the SAC305 micro-joints of flip chip assemblies during TS cycling, incorporating two influential factors of the static aging and stain-enhanced aging. The growth exponent (*n*) and strain-enhanced coarsening kinetics constant (*M*) were calculated as 1.70 and 3.49 × 10^6^ s, respectively. More significantly, the necking-coalescence was confirmed to be the predominant coarsening mode depending on the solute atoms transfer across the particle/solder matrix interface.

## Figures and Tables

**Table 1:** Material mechanical parameters [33].

Materials	Elastic Modulus (GPa)	CTE (10^−6^/°C)	Posson’s Ratio
Ni	200	13.4	0.31
Cu	120	17	0.35
Ag_3_Sn	74.5	20	0.35

**Table 2:** Energy-dispersive x-ray spectrometry (EDS) compositional analysis of intermetallic compounds (IMCs) phases formed in a Cu/SAC305/Ni micro-joint.

Analysis Sites	Composition (at.%)					Phase
	Ni	Cu	Sn	P	Ag	
Chip side	15.11	36.52	48.37	-	-	(Cu,Ni)_6_Sn_5_
Solder matrix	-	-	78.16	-	21.84	Ag_3_Sn
Substrate side	6.27	50.06	43.67	-	-	(Cu,Ni)_6_Sn_5_
